# Temperature-Dependent Menthol Binding across TRPM8 Conformational States

**DOI:** 10.1021/jacs.6c07735

**Published:** 2026-07-01

**Authors:** Leonardo Cirqueira, Guilherme Lucas, Carmen Domene

**Affiliations:** † Department of Chemistry, 1555 University of Bath, Bath BA2 7AX, United Kingdom; ‡ Department of Physiology, Ribeirão Preto School of Medicine, 67796 University of São Paulo, Ribeirão Preto 14049-900, Brazil

## Abstract

Cold sensation is mediated by TRPM8, a polymodal ion channel activated by temperature, pH, voltage, and cooling compounds such as menthol. Despite its central role in thermosensation and pharmacology, the molecular basis of menthol-mediated activation remains unclear, particularly how temperature influences ligand binding and channel activation. Polymodal ion channels further challenge site-centric views of ligand binding, as function emerges from coupled conformational equilibria and environment-dependent membrane partitioning. Here, we investigate temperature-dependent menthol distribution in TRPM8 using flooding molecular dynamics simulations of open and closed channel conformations across a physiological temperature range. This approach reveals distributed, low-affinity interactions that are not captured by discrete binding-site models. Below the TRPM8 activation threshold (<300 K), menthol preferentially accumulates in intracellular and interfacial regions, whereas above this threshold it redistributes toward transmembrane regions, indicating that temperature-dependent activation regimes reshape ligand partitioning at the protein–membrane interface. Across all conditions, menthol behaves as a low-affinity multisite ligand sampling a continuum of metastable interaction regions, consistent with experimentally identified interaction regions spanning the N-terminal domain, pore domain, voltage sensor-like domain (VSLD), and C-terminal region. A two-state allosteric model indicates that these temperature-dependent occupancy patterns shift the open–closed equilibrium of TRPM8. Together, these results suggest that temperature regulates function not only through channel energetics but also by modulating ligand interactions at the protein–membrane interface. This work identifies temperature-dependent ligand partitioning as a key physicochemical determinant of ligand efficacy in polymodal ion channels and provides molecular-level insight into menthol modulation of TRPM8.

## Graphical abstract

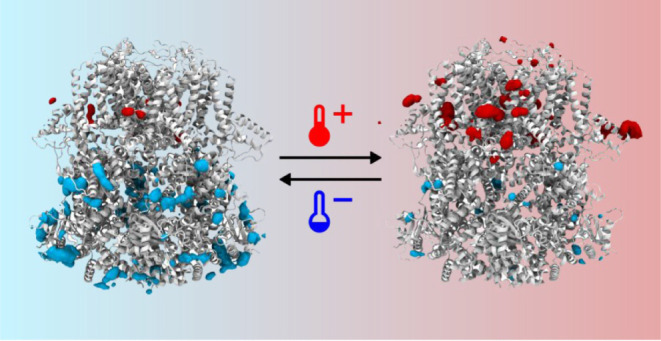

## Introduction

The ability to detect and respond to environmental stimuli is fundamental to physiology, allowing an organism to survive, maintain dynamic homeostasis, and adapt in a constantly changing environment. In vertebrates, dedicated molecular and cellular systems transduce physicochemical cues such as temperature, pressure, and pH into electrical signals that drive adaptive responses.^1^ The transient receptor potential melastatin 8 (TRPM8) is a nonselective cation channel that promotes Na^+^ and Ca^2+^ influx, leading to depolarization and increased excitability of sensory neurons at temperatures below 28 °C.^2,3^

Animal models lacking TRPM8 have established its canonical role in environmental cold detection.^4–6^ However, TRPM8 is also expressed in non-neuronal cells, where it plays a role in various physiological and pathological processes.^7,8^ Recent evidence suggests that TRPM8 regulates inflammatory and innate immune communication, linking it to tissue homeostasis and immune responses.^9–11^ Consistent with these roles, TRPM8 has been implicated in several pathologies, including urinary tract disorders,^12,13^ migraine,^14^ ocular disease,^15^ and cancer.^7,16^

The polymodal nature of TRPM8 is evident in its modulation by temperature, voltage, and chemical stimuli. TRPM8 is activated by natural cooling agents such as menthol,^2,3^ eucalyptol, and other terpenes,^17^ as well as synthetic compounds including icilin,^17^ WS-12,^18^ C3,^19^ and AITC.^20^ Endogenous regulators include phosphatidylinositol 4,5-bisphosphate (PI­(4,5)­P_2_—hereafter referred to as PIP_2_), an essential cofactor for channel activation.^21,22^ In addition, bacterial immunosuppressants such as rapamycin and tacrolimus have been reported to activate TRPM8.^23,24^ Conversely, TRPM8 is inhibited by several compounds,^25–28^ and channel activity is also downregulated by intracellular Ca^2+^, which promotes desensitization.^29^ Cold activation is further suppressed by acidic pH.^30^ Various analytical models have been proposed to explain the polymodal activation of TRPM8 by temperature, voltage, and ligands, differing in their assumptions regarding the number of states and the coupling between sensing mechanisms.^31–35^ Structural studies by cryo-EM have progressively resolved the molecular architecture of TRPM8 across diverse physicochemical conditions.^36–41^ The channel assembles as a homotetramer, with each subunit comprising six transmembrane helices (S1–S6), an interfacial TRP domain, and intracellular domains composed of melastatin homology regions MHR1–4 and the C-terminal region (Figure 1).

**Figure 1:**
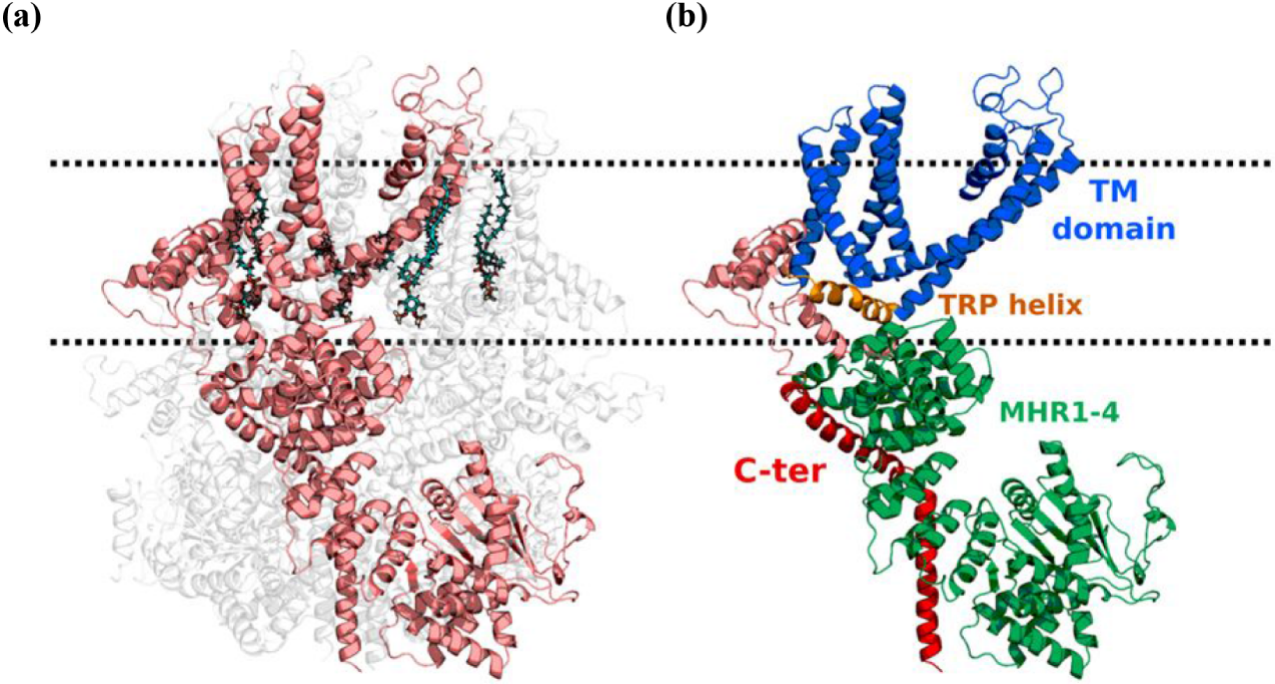
Structural overview of the open TRPM8 ion channel. The channel forms a tetrameric assembly (white), with one subunit highlighted in red. It comprises transmembrane (TM) and intracellular (IC) domains. PIP_2_ molecules reported in PDB ID 8E4L are shown as licorice representations. A single-subunit view highlights key structural elements: the transmembrane domain (blue), composed of six helices (S1–S6); the interfacial TRP helix (yellow); and the intracellular region, composed of the melastatin homology regions (MHR1–4, green) and the C-terminal tail (C-ter, red).

Electrophysiological studies have identified key determinants of menthol activation. Mutations primarily located in the transmembrane region impair TRPM8 activation by menthol.^42,43^ A notable example is the Y745H mutation in mouse TRPM8, which abolishes menthol sensitivity without affecting cold responses.^42^ This residue lies within the voltage-sensor-like domain (VSLD), which has been proposed as the primary binding site for menthol. This hypothesis is supported by experimental studies.^44,45^ Additionally, studies using menthol analogues and stereoisomers identified key pharmacophoric features for menthol interaction, including hydrogen bond donor/acceptor groups and hydrophobic moieties.^46,47^ More recently, high-resolution cryo-EM structures obtained at low temperatures have provided direct structural evidence, revealing that menthol occupies a binding pocket at the interface between the VSLD and the TRP domain.^36^

Despite these advances, contributions from other regions outside the VSLD may also play an important role in TRPM8 modulation. Mutations in other domains have been shown to significantly affect menthol sensitivity.^34,42,43,48–51^ The large internal cavities and structural heterogeneity of the fully assembled TRPM8 channel, combined with its polymodal regulation, complicate the characterization of menthol interactions across different regions, both experimentally and computationally. Conventional molecular dynamics (MD) simulations are often limited by insufficient sampling, particularly for low-affinity ligands such as menthol.^20,32,34,43^ Flooding (or cosolvent) MD simulations provide an alternative approach by increasing ligand concentration to enhance sampling and identify interaction regions across the protein surface.^52–54^ These approaches infer interaction preferences and estimate thermodynamic properties by analyzing ligand distributions.

Recent studies on membrane proteins, such as the Kv1.2 potassium channel, have shown that low-affinity interactions of small molecules can be described as degenerate interactions, where multiple regions exhibit similar affinities. This behavior can be interpreted using a partitioning framework, in which ligand thermodynamics are inferred from their distribution between bulk and the protein surface.^52^ Furthermore, the influence of ligands on conformational equilibria can be examined by comparing interaction patterns across different functional states.^55^ Given the complexity of TRPM8 gating and the multiple physicochemical factors that contribute to its activation, this framework is particularly well suited because it does not rely on the presence of a single high-affinity binding site. It captures distributed, low-affinity interactions across the protein surface that might otherwise be poorly characterized or detected. This approach has precedent in other membrane protein systems and is further supported by evidence that mutations outside the VSLD modulate menthol sensitivity.^42,48,50,56^

Although temperature is the primary driver of TRPM8 activation, the modulation of menthol activity across temperature ranges remains poorly understood. Temperature modulates the hydrophobic effect, thereby influencing ligand partitioning and interaction patterns. Experimental evidence indicates menthol activation of TRPM8 is enhanced at lower temperatures,^2,3^ suggesting a synergistic interplay between thermal and chemical stimuli. While distinct domains have been demonstrated for sensing cold and menthol,^27,57^ their effects converge on shared activation pathways. Elucidating how temperature modulates menthol action has emerged as an important and relevant question, with implications for understanding the diverse modes of TRPM8 regulation.

To address how temperature shapes menthol-TRPM8 interactions, we employ flooding MD simulations across 279–310 K on both open and closed channel conformations. Menthol behavior is interpreted within a partitioning framework that accounts for distributed, low-affinity interactions across the protein–membrane interface, an approach particularly suited to ligands that do not operate through a single well-defined binding site. Our results support a model in which menthol activation of TRPM8 arises from temperature-dependent partitioning over multiple sites rather than being solely dominated by a single high-affinity binding event.

## Methods

### Ligand Partitioning Framework

Menthol is a small, naturally occurring monoterpene that combines a hydrophobic cyclohexane scaffold with a hydroxyl group, conferring amphiphilic character and a tendency to partition between aqueous and hydrophobic environments. Unlike classical drugs that engage a single high-affinity binding pocket through specific directional interactions, menthol’s physicochemical properties predispose it to distributed, low-affinity interactions across multiple regions of the protein surface. This behavior can be more formally described as a partitioning process between the protein surface and the bulk solvent^52^ and allows us to define a volume ν as a continuous phase of homogeneous affinity within which menthol interactions occur in a degenerate manner. Under low-occupancy conditions, where interactions are weak and dominated by translational degrees of freedom, the aggregate equilibrium constant for the reaction P_0_ + nL → PL_n_ (where P denotes the protein and L the ligand) can be expressed in terms of the partition coefficient p , which can be determined from flooding simulations:

$$p=\frac{\left\langle n\right\rangle }{\nu }\times {\left(\frac{N-n}{V-\nu }\right)}^{-1}$$

where V is the total system volume containing N indistinguishable menthol ligands. The protein surface volume ν was defined as the region within 5 Å of the protein atoms. The volume was calculated using MSMS^58^ as the difference between (i) the volume of the protein expanded by a 5 Å shell and (ii) the volume of the protein alone. Both calculations employed a probe radius of 1.4.^52^ The partition number or occupancy, ⟨n⟩, is defined as the average number of ligands present within the volume ν at equilibrium.

The concentration invariance of the partition coefficient allows extrapolation across ligand concentrations using theoretical projections.^52^ A partition coefficient obtained from high-concentration flooding MD $(p^{\prime }$ can be used to predict properties at other ligand concentrations, including titration curves, free energy (ΔW), and the three-dimensional spatial distribution (ρ­(R)) respectively:

$$\left\langle n\right\rangle ={\left(1+\frac{V-\nu }{\nu p^{\prime }}\right)}^{-1}N$$

$$\Delta W=-\beta^{-1}\ln\left(\frac{N^{\langle n\rangle }}{\left\langle n\right\rangle !}\right)-\beta^{-1}\left\langle n\right\rangle \ln\left(\frac{V-\nu }{\nu p^{\prime }}\right)$$

$$\rho \left(R\right)=\left\langle n\right\rangle \rho^{\prime }\left(R\right)$$

where *R* is the Cartesian position of each ligand and $\rho^{\prime }\left(R\right)$ is the unitary ligand distribution, normalized such that $\int \rho^{\prime }\left(R\right)dR=1.$

### Allosteric Modulation and Voltage-Dependent Gating

Voltage activation of ion channels can be macroscopically described as an equilibrium between two states, closed and open, driven by a transmembrane voltage ΔV. The probability that a channel opens in response to an electrical stimulus (P_O_) is described by the two-state Boltzmann equation, with the voltage midpoint (*V*_1/2_) and the gating charge (ΔQ) as defining parameters:

$$P_{O}\left(\Delta V\right)={[1+e^{+\beta \left(V_{1/2}-\Delta V\right)\Delta Q}]}^{-1}$$

In the presence of both voltage and ligand modulation, the two-state Boltzmann equation includes an additional ligand-affinity parameter, α:^55,59^

$$P_{O}\left(\Delta V\right)={[1+\alpha e^{+\beta \left(V_{1/2}-\Delta V\right)\Delta Q}]}^{-1}$$

Here,

$$\alpha =\frac{Z_{C}}{Z_{O}}\times e^{+\beta \cdot \Delta V\cdot \Delta \Delta Q}$$

represents the ratio of grand-canonical partition functions for the closed (C) and open (O) states, including contributions from changes in gating charge (ΔΔQ = ΔQ_C_ – ΔQ_O_) upon ligand binding. However, electrophysiological measures indicate that menthol does not significantly alter the gating charges of TRPM8,^43^ implying ΔΔQ = 0. Under this condition, the expression reduces to a ligand-dependent partitioning term:

$$\alpha =\frac{Z_{C}}{Z_{O}}$$

Preferential stabilization of the open state corresponds to α < 1, producing a leftward shift in the voltage–activation curve, whereas α > 1 indicates preferential stabilization of the closed state. When ligand interactions are conformation-independent, α = 1 and the system recovers the classical two-state Boltzmann model. For the case of low-affinity, degenerate interactions, the partition function can be expressed in terms of the ligand concentration ([L]), the partition coefficient p and partition number ⟨n⟩ for each conformational state:^55^

$$\frac{Z_{C}}{Z_{O}}=\frac{1+\frac{{\left([L]\nu p_{C}\right)}^{\left\langle n_{C}\right\rangle }}{\langle n_{C}\rangle !}}{1+\frac{{\left([L]\nu p_{O}\right)}^{\left\langle n_{O}\right\rangle }}{\langle n_{O}\rangle !}}$$

### Coarse-Grained (CG) Simulations

Atomic coordinates for open and closed TRPM8 conformations were retrieved from the Protein Data Bank (PDB IDs 8E4L and 8E4N, respectively).^40^ The open structure (8E4L) was solved in the presence of PIP_2_ and the agonists C3 and AITC, whereas the closed structure (8E4N) was determined in the presence of PIP_2_ only. Missing residues were modeled using ColabFold,^60^ with default MSA parameters (*msa_mode: mmseqs2_uniref_env*, *pair_mode: unpaired_paired*, and *template_mode: custom*), using the corresponding cryo-EM structures as templates. The pre-MHR region was removed. Initial open and closed conformations of TRPM8 were converted into CG models using *Martinize2*.^61^ Protein structures were generated with DSSP-based secondary structure restraints^62,63^ applied to the backbone, and an elastic network model with lower and upper cutoffs of 0 and 8.5 Å, respectively. The options “*-ignh*”and “*-cys none*” were also applied. The INSANE tool,^64^ updated for Martini 3 lipidome,^65^ was used to embed the transmembrane region of TRPM8 into a POPC bilayer. Four CG PIP_2_ molecules (modeled as SAP6 in the Martini 3 lipidome) were manually positioned according to the PIP_2_-binding sites observed in the corresponding cryo-EM structures of TRPM8. To assess the effect of PIP_2_, additional systems were constructed using symmetric bilayers composed exclusively of POPC, lacking PIP_2_. The system was subsequently solvated using INSANE in a simulation box of 180 × 180 × 200 Å; ions were added to achieve a 150 mM NaCl concentration. Menthol molecules were then progressively inserted at random positions in the aqueous phase to a final concentration of 10 mM (37 molecules). Simulations were performed using GROMACS 2025.2 with the Martini 3 force field for protein, lipids, water, and ions. Nonbonded interactions were computed with an 11 Å cutoff. Temperature was maintained using the V-rescale thermostat.^66^ CG simulations were initiated with 2000 steps of energy minimization, followed by 1,000,000 steps of equilibration under 1 atm pressure controlled by a Berendsen barostat.^67^ Production runs were carried out for 3000 ns (1.5 × 10^8^ steps), with pressure maintained at 1 atm using a Parrinello–Rahman barostat.^68^ A 20 fs integration time step was employed for both equilibration and production runs. Each conformational state was simulated in triplicate at four temperatures: 279 K (∼5.9 °C), 290 K (∼16.9 °C), 300 K (∼26.9 °C), and 310 K (∼36.9 °C). Analysis was performed using in-house scripts in VMD^69^ and data were restricted to production trajectories. A summary of the simulations performed in this study is reported in Table 1.

**Table 1:** Summary of CG TRPM8-Menthol Flooding Simulations^a^

Temperature (K)	Conformation	With PIP_2_ (ns)	Without PIP_2_ (ns)
279 (∼6 °C)	Open	3 × 3000	3 × 3000
	Closed	3 × 3000	3 × 3000
290 (∼17 °C)	Open	3 × 3000	3 × 3000
	Closed	3 × 3000	3 × 3000
300 (∼27 °C)	Open	3 × 3000	3 × 3000
	Closed	3 × 3000	3 × 3000
310 (∼37 °C)	Open	3 × 3000	3 × 3000
	Closed	3 × 3000	3 × 3000

a Simulations were performed for open and closed conformations across four temperatures (279, 290, 300, and 310 K), with and without PIP_2_ in the lower leaflet. Each condition was simulated in triplicate, with a production length of 3000 ns per replicate.

### CG Model Development for the Ligand

Atom-to-bead mapping is a critical step in CG ligand model development. In this study, menthol was mapped from 31 atoms into four beads, reproducing key physicochemical properties.^70^ Adaptation to the Martini 3 force field required dedicated parametrization using the Martini Fast_Forward workflow,^71,72^ which integrates all-atom and CG system preparation, parameter derivation, and validation. The all-atom reference system consisted of a single menthol molecule solvated in 2606 water molecules with 150 mM NaCl. Simulations were performed in GROMACS 2021.4 ^73^ with 50,000 energy minimization steps, 500,000 equilibration steps, and 5,000,000 production steps using a 2 fs time step and a 12 Å cutoff. Temperature was maintained at 300 K with a V-rescale thermostat, while pressure (1 atm) was controlled using the C-rescale barostat^74^ during equilibration and the Parrinello–Rahman barostat during production. Initial CG parameters were derived using the Fast_Forward “*ff_inter module*”, which maps all-atom trajectories onto the CG representation to derive bonded interactions. Due to the geometry of menthol, suggested improper terms and constraints were omitted. The CG model was validated using a single menthol molecule solvated in 478 CG water beads with 150 mM NaCl. Simulations were performed with an 11 Å cutoff and the same V-rescale thermostat at 300 K. The system was energy-minimized for 200,000 steps, equilibrated for 1,000,000 steps (10 fs time step, C-rescale barostat at 1 atm), followed by 5,000,000 production steps (20 fs time step; 100 ns total) using the Parrinello–Rahman barostat. Model quality was assessed with the Fast_Forward “*ff_assess”* module by comparing structural and energetic properties (*e.g.,* bond lengths and angles) between all atom and CG representations. Parameters were iteratively refined until satisfactory agreement with the all-atom reference was achieved.

## Results

Flooding MD simulations across 279–310 K reveal a clear temperature dependence in menthol partitioning across the TRPM8 surface. All systems remained stable throughout the production trajectories (Figure S2), with no major conformational changes observed, as expected given the rigidity of the elastic network model and the accessible simulation time scales. RMSD profiles also showed no significant structural differences between TRPM8 CG models with and without PIP_2_. Menthol molecules distributed across all systems, with increasing preference for hydrophobic environments at higher temperatures (Figures 2 and S3).

**Figure 2:**
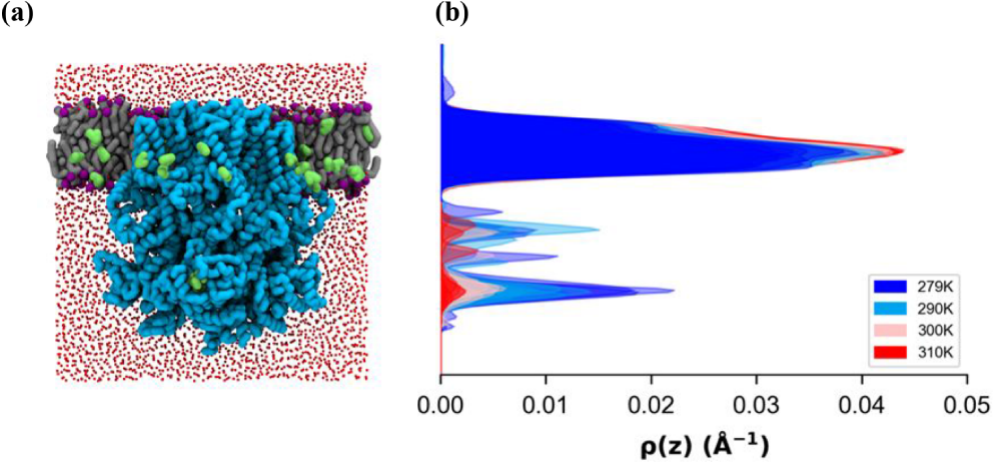
Coarse-grained (CG) system overview. (a) CG representation of the open TRPM8 ion channel (blue) embedded in a gray bilayer, and with menthol molecules in green and the aqueous solvent in red. (b) Menthol density profiles along the membrane normal (*z*-axis) showing ligand distributions relative to the membrane at 279, 290, 300, and 310 K. The *z*-axis in panel (b) follows the same vertical scale as that shown in panel (a).

Quantitative equilibrium partition properties of menthol–TRPM8 interactions across temperatures are summarized in Table 2. Sampling remained homogeneous among monomers, with residue-specific contact deviations averaging below 5%. Total ligand occupancy decreased by approximately 50% from 279 to 310 K, with the difference arising predominantly from interactions with the intracellular domain, which declined steadily with increasing temperature. Interactions with the transmembrane domain, by contrast, remained largely unchanged across all conditions. At lower temperatures (279 and 290 K), the open conformation exhibited consistently higher ligand occupancy and partition coefficients than the closed state, consistent with ligand-mediated channel activation. At higher temperatures (300 and 310 K), partition coefficients for both conformations became nearly identical, indicating a progressive weakening of menthol modulation. This behavior was observed regardless of the presence of PIP_2_, suggesting that inclusion of PIP_2_ does not substantially alter the overall ligand partitioning behavior of the protein models. The partition coefficient is directly related to the average partition number ⟨n⟩; accordingly, convergence was primarily assessed from the time evolution of this quantity, shown as black traces in Figure S3. Across all replicas, ⟨n⟩ fluctuated around stable mean values without systematic drift, supporting convergence of the calculated partition properties during the production simulations. In addition, the fractions of menthol molecules partitioned into the lipid and aqueous phases remained stable throughout the trajectories.

**Table 2:** Partition Properties at Equilibrium from CG TRPM8-Menthol Flooding MD Simulations for Open and Closed Conformations, with and without PIP_2_ (N = 37 Menthol Molecules, 10 mM)^a^

Temperature	PIP_2_ presence	Conformation	V/10^4^ Å^3^	ν/10^4^ Å^3^	⟨n_TM_⟩	⟨n_IC_⟩	⟨n⟩	p	Open/Closed ratio
279 K (6 °C)	With PIP_2_	Open	504 ± 1	57 ± 0	2.4 ± 0.4	10.1 ± 1.6	12.5 ± 1.3	4.0 ± 0.7	1.2 ± 0.2
		Closed	504 ± 0	57 ± 0	2.5 ± 0.1	8.4 ± 0.6	10.9 ± 0.4	3.3 ± 0.2	
	Without PIP_2_	Open	507 ± 1	56 ± 0	2.2 ± 0.1	12.1 ± 0.9	14.4 ± 0.7	5.1 ± 0.4	1.5 ± 0.2
		Closed	505 ± 1	56 ± 1	2.1 ± 0.1	8.8 ± 0.8	10.9 ± 0.9	3.3 ± 0.4	
290 K (17 °C)	With PIP_2_	Open	509 ± 1	57 ± 0	2.2 ± 0.2	9.5 ± 0.7	11.7 ± 0.7	3.7 ± 0.3	1.3 ± 0.2
		Closed	509 ± 0	57 ± 0	2.4 ± 0.4	7.5 ± 0.6	9.9 ± 0.7	2.9 ± 0.3	
	Without PIP_2_	Open	511 ± 1	56 ± 0	1.8 ± 0.1	10.8 ± 1.7	12.6 ± 1.5	4.2 ± 0.8	1.4 ± 0.3
		Closed	510 ± 0	56 ± 1	1.9 ± 0.1	8.1 ± 1.0	10.0 ± 1.0	3.0 ± 0.4	
300 K (27 °C)	With PIP_2_	Open	513 ± 0	56 ± 1	2.1 ± 0.2	6.2 ± 0.7	8.2 ± 0.9	2.4 ± 0.4	1.0 ± 0.2
		Closed	513 ± 0	56 ± 1	1.9 ± 0.1	6.3 ± 0.5	8.2 ± 0.5	2.3 ± 0.2	
	Without PIP_2_	Open	516 ± 0	56 ± 1	2.2 ± 0.1	6.7 ± 0.5	8.8 ± 0.6	2.6 ± 0.3	1.0 ± 0.1
		Closed	515 ± 0	56 ± 0	2.1 ± 0.2	6.9 ± 0.5	9.0 ± 0.3	2.6 ± 0.1	
310 K (37 °C)	With PIP_2_	Open	518 ± 1	55 ± 0	2.0 ± 0.1	5.7 ± 0.4	7.7 ± 0.4	2.2 ± 0.2	1.1 ± 0.1
		Closed	517 ± 0	55 ± 0	1.7 ± 0.0	5.4 ± 0.1	7.1 ± 0.1	2.0 ± 0.1	
	Without PIP_2_	Open	524 ± 3	55 ± 1	1.8 ± 0.1	5.7 ± 0.7	7.5 ± 0.7	2.2 ± 0.3	1.0 ± 0.1
		Closed	520 ± 0	55 ± 0	2.0 ± 0.1	5.8 ± 0.2	7.7 ± 0.2	2.2 ± 0.1	

a Each condition was simulated in triplicate (2000 steps minimization, 20 ns equilibration, 3000 ns production). The average number of interacting ligands, ⟨n⟩, was computed using a 5 Å proximity cutoff for transmembrane (TM) and intracellular (IC) segments separately. Total volume (V) was obtained from periodic cell dimensions; site volume (ν) was calculated using MSMS from the all-atom representation at trajectory end points. Open/closed ratios were estimated by error propagation.

Spatial density analysis revealed that menthol sampled a broad interaction landscape across the TRPM8 surface, spanning the pore domain, VSLD, and intracellular domains (Figure 3). Cluster analysis identified more than 20 distinct binding regions in each density map, with comparable densities across all temperature conditions and reversible binding observed throughout the simulations. This pattern was consistent across both open and closed conformations, reinforcing that the multisite interaction mode is a robust feature of menthol-TRPM8 interactions rather than a conformation-specific artifact. Overall, ligand sampling patterns were qualitatively similar across the different subunits, although local variations in occupancy were observed, as expected for stochastic behavior. Consistent with the homogeneous distribution observed across subunits, the density representation shown for a single subunit in Figure 3 is representative of the shared interaction regions across the tetramer. Accordingly, the densities were symmetrized onto the full tetrameric assembly to emphasize the common interaction landscape, while local differences in occupancy remained consistent with stochastic ligand behavior.

**Figure 3:**
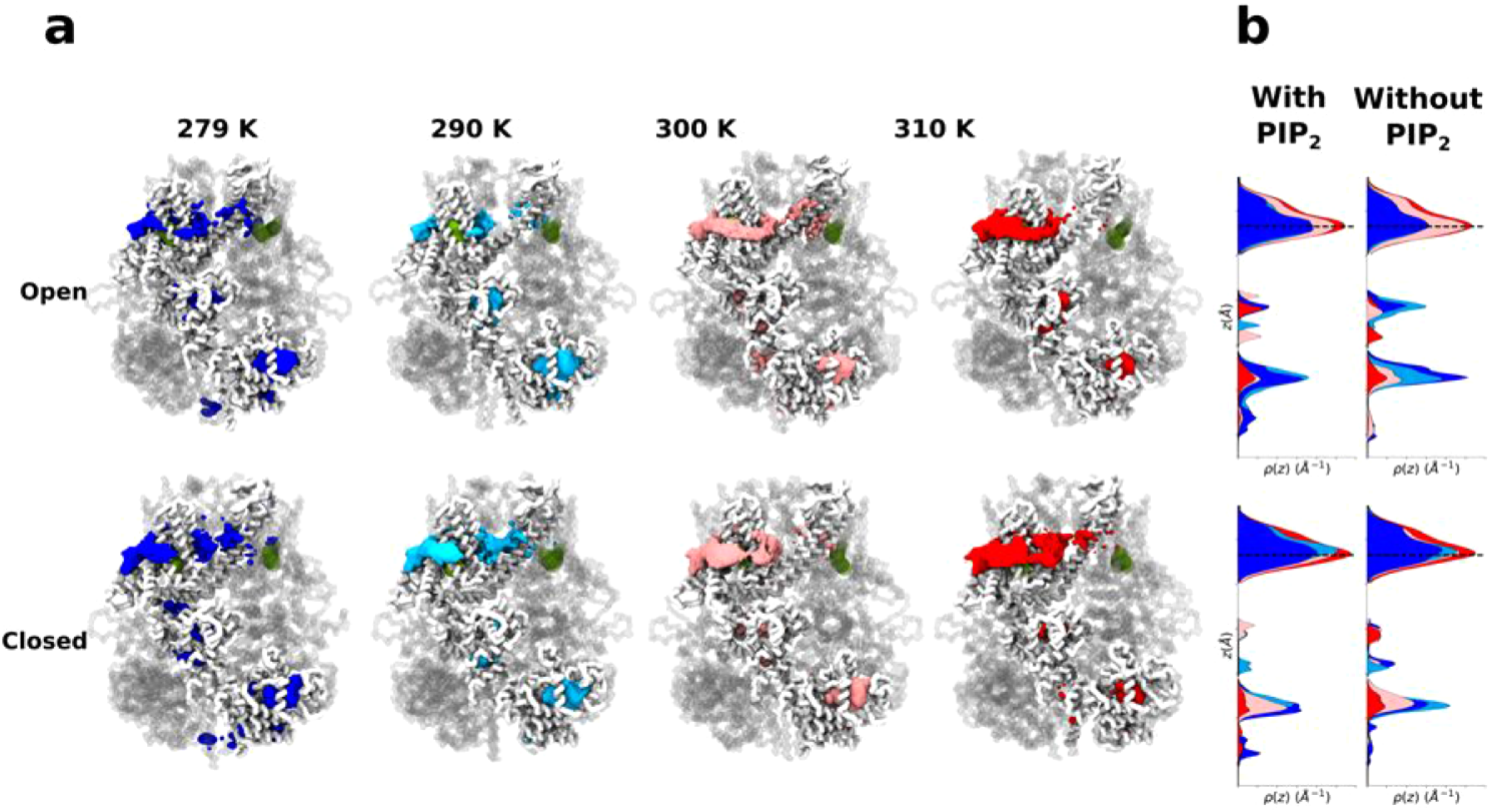
Spatial density maps of menthol obtained from CG flooding simulations. (a) Merged spatial density maps were generated by combining trajectories performed in both the presence and absence of PIP_2_. Because sampling remained homogeneous across all four subunits, menthol densities were symmetrized and projected onto the full tetrameric assembly as isosurfaces at an isovalue of 3.2 × 10^–5^ Å^–3^ (∼53 mM). The full tetrameric assembly is shown in transparent representation, with one subunit highlighted for clarity. Residue Y745 is highlighted in green. Clustering analysis identified more than 20 contiguous regions per map, supporting the presence of multiple distributed interaction sites across the protein surface. Temperature is color-coded as follows: 279 K (light blue), 290 K (blue), 300 K (pink), and 310 K (red). (b) One-dimensional density profiles along the membrane normal for simulations performed in the presence and absence of PIP_2_. The *z*-axis follows the same scale as in panel (a), whereas the dashed line represents the average vertical position of residue Y745.

Despite the well-established role of Y745 in menthol sensitivity, where its mutation abolishes menthol activation without affecting cold responses, menthol contact occupancy at Y745 remained below 3% under all conditions examined (Table S2). Other residues identified in high-resolution structural studies as important for menthol interaction (R842, Y1005, R1008, and F1013)^36^ similarly exhibited low contact frequencies throughout the simulations, except for F1013, which displayed higher interaction frequencies of 3.7% ± 1.6% and 4.5% ± 2.5% in the open and closed conformations, respectively (Figure S4). This increased sampling may be related to the greater solvent exposure of F1013, whereas the remaining residues are more deeply buried within the VSLD pocket and are therefore less accessible within the time scales and structural constraints of the present CG simulations. In contrast, several residues previously associated with TRPM8 modulation in mutagenesis studies, but located outside the structurally resolved orthosteric pocket,^42,48,50^ displayed substantially higher interaction frequencies than Y745. These included residues from the N-terminal region (L509, F523, V524, W525, L527, V528, and F531), the pore region (V879 and Y908), the peripheral region of VSLD (T840) and the C-terminal region (F1021) (Figure S5).

Most interactions occurred outside the voltage sensor-like domain and away from the proposed orthosteric pocket between the TRP helix and VSLD, indicating that this primary site is rarely sampled on the time scales accessible to the present simulations (Figures S4 and S6), despite experimental evidence supporting its functional relevance. This apparent discrepancy likely reflects differences between structurally resolved ligand-bound states and the broader ensemble of transient interactions captured in flooding simulations. Taken together, these observations are consistent with a degenerate, multisite binding mode in which distributed regions of the protein surface collectively dominate the interaction landscape. Although the canonical orthosteric site is known to be relevant, its relatively low sampling within the simulated time scales for all trajectories resulted in a small contribution to the total menthol occupancy.

The higher partition numbers and coefficients observed for the open state at 279 and 290 K are consistent with ligand-mediated activation in two-state models,^55,59^ where preferential ligand association with the open conformation thermodynamically biases the channel toward activation. This result provides a molecular-level rationalization of the macroscopic observation that menthol potentiation of TRPM8 is enhanced at lower temperatures. Using the partition coefficients derived for both conformations, it is possible to reconstruct the voltage-dependent open probability (P_O_) of the channel under the combined influence of voltage and menthol, within the two-state allosteric framework (Figure 4).

**Figure 4:**
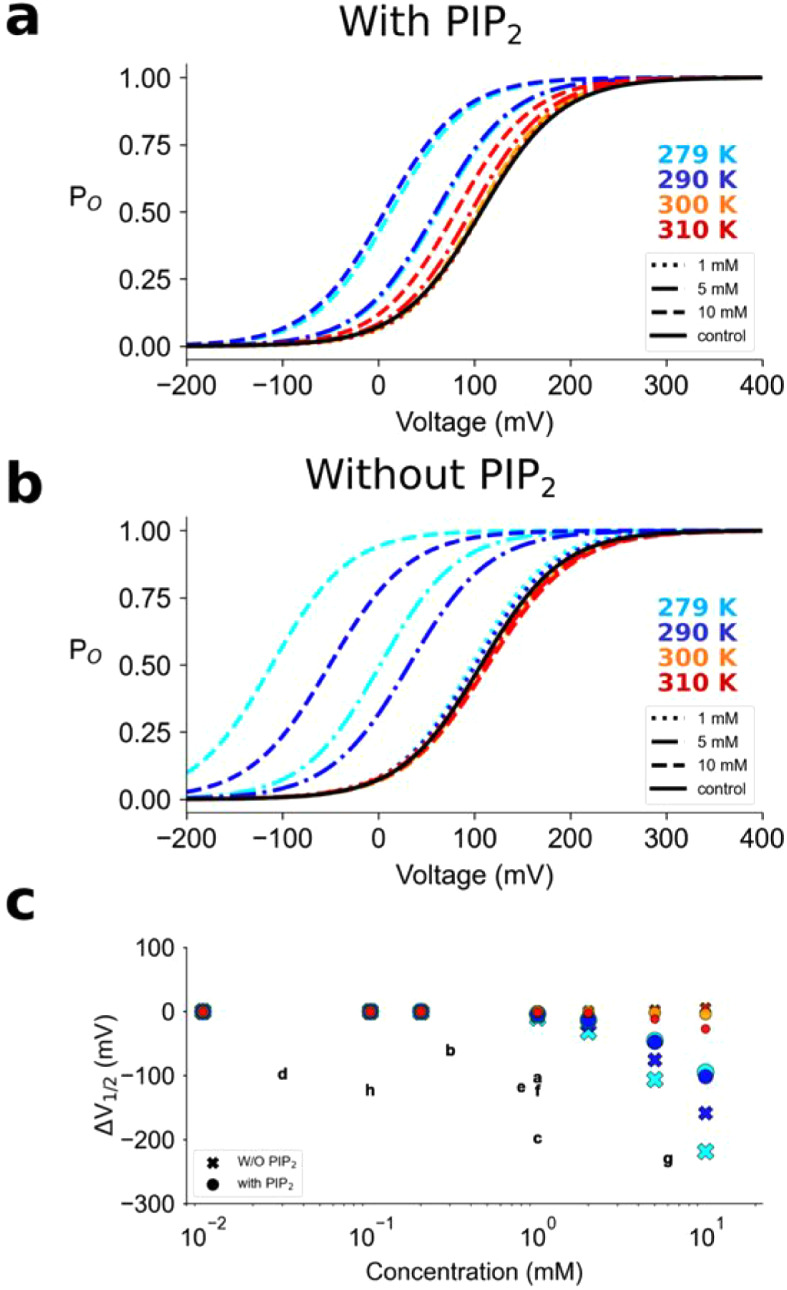
Theoretical projections from two-state allosteric models of TRPM8–menthol interaction. Voltage-activation curves (P_O_) predicted from CG simulations with (a) and without (b) PIP_2_ reconstructed using a two-state Boltzmann model with experimental reference parameters from *Mus musculus* electrophysiology^40^ (ΔQ = 0.6 e, V_1/2_ = −107 mV). Partition coefficients were combined with ligand concentration to define the conformational affinity parameter α.^52,55^ Line styles denote menthol concentration; colors denote temperature (279 K, light blue; 290 K, blue; 300 K, orange; 310 K, red). The control curve is shown in black. (c) Predicted voltage shift (ΔV_1/2_) as a function of menthol concentration, for simulations with PIP_2_ (circles) and without PIP_2_ (crosses). Alphabetical labels indicate experimental ΔV_1/2_ values from electrophysiology studies: (a) Taberner et al., 2014,^34^ *Rattus norvegicus*, 30 °C; (b) Bandell et al., 2006,^42^ *Mus musculus*, 26 °C; (c) Voets et al., 2004,^35^ *Homo sapiens*, 34 °C, and Voets et al., 2007,^43^ *Homo sapiens*, 25 °C; (d) Arcas et al., 2024,^24^ *Mus musculus*, 33 °C; (e) Arcas et al., 2019,^23^ *Mus musculus*, 24 °C; (f) Janssens et al., 2011,^32^ *Homo sapiens*, 25 °C; (g) Matta et al., 2007,^33^ *Rattus norvegicus*, 25 °C; (h) Malkia et al., 2009,^27^ *Mus musculus*, 33 °C.

At lower temperatures, the larger difference in partition coefficients between open and closed conformations drives a leftward shift in the voltage-activation curve, consistent with enhanced TRPM8 activation by menthol at 279 K. At 310 K, partition coefficients for both conformations converge, and no significant shift is predicted, consistent with the attenuation of menthol modulation above the activation threshold. The predicted leftward shift becomes more pronounced with increasing menthol concentration in both the PIP_2_ and non-PIP_2_ models, with ΔV_1/2_ values falling within the same order of magnitude as experimental measurements (Figure 4). This concentration dependence supports an allosteric mechanism in which small conformational differences in ligand affinity become amplified at higher ligand occupancy, collectively producing a significant bias toward channel opening.

## Discussion

TRPM8 is a polymodal ion channel primarily involved in cold sensation and is well-known for its activation by cooling agents, particularly monoterpenes such as menthol. This functional diversity makes TRPM8 an important pharmacological target and a challenging system for understanding the molecular basis of temperature sensing. In this study, we investigated the molecular features of TRPM8 modulation by menthol using flooding MD simulations performed at temperatures within and beyond the channel activation range.^2,3^ The availability of structural data for both open and closed conformations of TRPM8 ^40^ enabled independent simulations of each state, allowing direct comparison of their interaction profiles with menthol. These simulations revealed clear temperature-dependent differences in menthol–TRPM8 interactions, with stronger association observed at lower temperatures.

Temperature strongly modulates hydrophobic and solvation-driven effects, which underpin the observed redistribution of menthol between protein and membrane environments. At lower temperatures, menthol preferentially accumulates in intracellular and interfacial regions of TRPM8, whereas at higher temperatures it shifts toward more membrane-embedded environments. These trends are consistent with temperature-dependent changes in solvation free energy and lipid partitioning. Importantly, hydrogen–deuterium exchange experiments have shown that cold sensitivity in TRPM8 is primarily thermodynamic in origin,^36^ with key contributions from the pore helix and lipid interactions, particularly PIP_2_, which stabilizes conductive states at lower temperatures. Our results are consistent with this thermodynamic framework, suggesting that temperature-dependent ligand redistribution contributes to the same energetic landscape that governs channel activation.^44^

A well-established menthol interaction site is in the voltage-sensor-like domain (VSLD), near residue Y745, where mutagenesis studies have shown that disruption of this region abolishes menthol sensitivity. Structural and computational studies have further supported this region as a key orthosteric site. However, in the present simulations, this region was only sparsely sampled, indicating that stable binding events at the canonical VSLD pocket are not prominently captured within the coarse-grained flooding framework. Instead, menthol interactions are dominated by transient, distributed contacts across the protein–membrane interface, suggesting that multiple weak interaction sites contribute to the observed behavior.

Quantitative analysis shows that at lower temperatures (279–290 K), menthol occupancy is comparable across transmembrane regions in both open and closed conformations, while intracellular interactions are enhanced in the open state. This asymmetry leads to distinct partition coefficients between conformations and produces a thermodynamic bias favoring channel opening. At higher temperatures (300–310 K), intracellular interactions decrease substantially, and partition coefficients converge between conformations, resulting in minimal predicted modulation of channel gating. These changes are consistent with a temperature-dependent shift in menthol partitioning between hydrophilic and hydrophobic environments and align with experimentally observed reductions in menthol efficacy at elevated temperatures.^2,3,42,45^

Several features of the CG flooding approach, including elastic-network restraints, elevated ligand concentrations, and frequent multiligand encounters, limit the ability to resolve detailed allosteric pathways or rare binding events. While the model captures large-scale redistribution of menthol across the protein–membrane interface, it does not fully resolve stereospecific interactions or capture stable occupancy of low-probability binding sites such as the VSLD pocket. These limitations highlight the need for higher-resolution approaches to fully characterize orthosteric binding behavior.

Taken together, our results support a model in which menthol modulation of TRPM8 arises from both discrete orthosteric interactions at the VSLD and distributed low-affinity interactions across the protein–membrane interface (Figure 5). However, the present simulations predominantly capture the latter regime, with limited sampling of the canonical binding site. We therefore propose that temperature-dependent ligand partitioning acts as an additional regulatory layer influencing TRPM8 modulation by controlling the availability of ligand to both orthosteric and distributed interaction sites. More broadly, these findings suggest that small-molecule modulation of polymodal ion channels is governed not only by specific binding events but also by temperature-dependent redistribution within the membrane environment.

**Figure 5:**
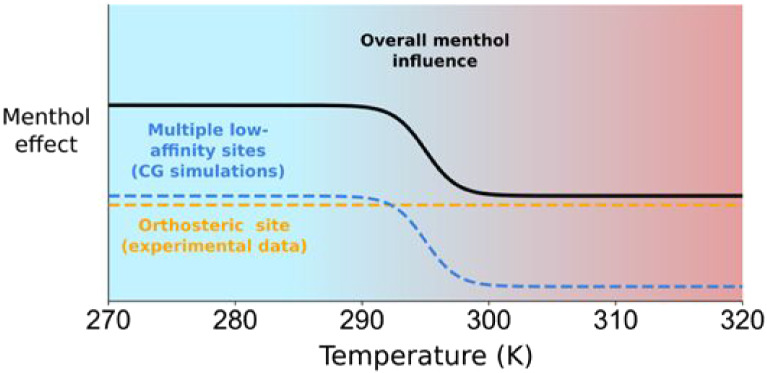
Schematic representation of a proposed mechanism for temperature-dependent menthol modulation of TRPM8. Axis scales are arbitrary and do not reflect quantitative measurements, as the relative contributions of each component could not be determined in the present study. The overall menthol effect (black solid line) is hypothesized to arise from two components: (i) the canonical orthosteric site near residue Y745 (orange dashed line), supported by previous experimental and structural studies but not sampled in the present CG simulations, and (ii) distributed low-affinity interaction sites across the protein surface, particularly within intracellular regions (blue dashed line), as characterized in the current work. Increasing temperature is proposed to markedly reduce the contribution from the distributed low-affinity component, likely driven by temperature-dependent changes in ligand partitioning and the hydrophobic effect. The temperature dependence of the orthosteric contribution remains speculative in this context and is depicted here as constant solely for illustrative purposes. The combined effect would result in reduced overall menthol modulation at physiologically relevant temperatures (>25 °C).

## Conclusions

The question of how cold sensation arises at the molecular level has motivated decades of research into the biophysics of thermosensitive ion channels. TRPM8 has emerged as the principal molecular detector of environmental cold, yet the mechanisms by which thermal and chemical stimuli cooperate to regulate its gating remain incompletely understood. The polymodal nature of TRPM8 presents inherent challenges for disentangling the contributions of individual stimuli, particularly in experimental settings. This coupling problem is representative of a broader class of polymodal ion channels in which multiple physical inputs converge on overlapping structural elements. Resolving stimulus-specific contributions therefore requires ensemble-based descriptions of ligand–protein–membrane energetics rather than static structural interpretations. Here, we apply a computational partitioning framework to both open and closed conformations of TRPM8 across a range of temperatures, isolating temperature-dependent effects on menthol–channel interactions and providing mechanistic insight into stimulus coupling.

The results show that temperature directly modulates menthol interaction patterns: at lower temperatures (279 and 290 K), interactions with intracellular and cytosolic regions are enhanced, whereas at higher temperatures (300 and 310 K), menthol preferentially partitions toward the transmembrane environment. Although the proposed orthosteric site near Y745 is strongly supported by experimental evidence,^36^ it is rarely sampled within the accessible time scales of the present simulations. Instead, multiple regions across the protein surface exhibit substantial ligand occupancy, particularly within intracellular domains. Projections to physiologically relevant conditions therefore support a view in which menthol interactions are better described as a low-affinity, multisite partitioning process that complements binding to discrete high-affinity sites.

CG simulations were essential for accessing the time scales required to resolve these distributed interactions, enabling characterization of spatially heterogeneous, low-affinity contacts that are difficult to capture at higher resolution. This highlights a limitation of binding-site-centric interpretations: rare but functionally relevant ligand configurations may be systematically under-sampled when analysis is restricted to a small number of high-affinity minima. In contrast, partition-based sampling reveals a continuum of metastable interaction states whose collective occupancy is more directly relevant to modulation. Within this framework, menthol effects arise from spatially distributed interactions that are strongly temperature-dependent and consistent with coupling to channel gating.

At a more fundamental level, ligand behavior in this regime is better described in terms of spatially resolved chemical potential gradients across heterogeneous protein–lipid environments rather than discrete binding equilibria alone. In this thermodynamic framing, binding sites emerge as local hotspots within a broader partitioning landscape. This interpretation explains the observed temperature dependence: enhanced intracellular interactions at lower temperatures preferentially stabilize the open state, whereas these interactions are weakened at higher temperatures in both conformations, eliminating preferential stabilization. This behavior is captured by the two-state allosteric model, in which parameters derived below 290 K reproduce leftward shifts in channel activation, whereas higher-temperature parameters approximate control conditions. Importantly, these results suggest that temperature modulates not only protein conformational energetics but also ligand spatial distribution, thereby reshaping the effective allosteric landscape experienced by the channel.

More broadly, this work supports a general framework in which small hydrophobic ligands modulate membrane proteins through concentration-dependent partitioning across the protein–membrane interface.^52,55^ In this view, functional outcomes arise from ensembles of distributed interactions rather than single binding events. From a drug discovery perspective, this implies that optimizing ligand efficacy for membrane protein targets may require controlling not only affinity for specific binding pockets, but also global partitioning behavior within the membrane–protein. This may be particularly relevant for hydrophobic or amphipathic ligands targeting polymodal receptors, where efficacy emerges from distributed occupancy rather than canonical lock-and-key binding. More generally, ligand function in membrane-embedded proteins arises from environment-dependent interaction ensembles in which temperature, membrane composition, and protein conformational state jointly define the pharmacological landscape. This framework provides a generalizable paradigm for understanding and designing modulators of polymodal membrane proteins, in which environmental conditions influence not only protein conformation but also ligand distribution across multiple interacting regions. Importantly, while the present results support this partition-based mechanism, the relative contribution of distributed interactions versus the canonical Y745/VSLD orthosteric site remains unresolved due to limited sampling of that region in the current CG simulations. Further high-resolution simulations and targeted studies will therefore be required to fully disentangle these contributions.

Ultimately, this work highlights a broader view of small-molecule modulation in which classical single-site binding is complemented by a thermodynamically governed landscape of distributed, environmentally sensitive interactions that collectively shape channel function.

## Supplementary Material



## Data Availability

A compressed archive is available at 10.5281/zenodo.19826646 containing: (1) CG menthol model and parametrization file as reported in the Supporting Information; (2) PDB files for CG simulation systems of TRPM8 in open and closed conformations, with and without PIP_2_; (3) representative CG protein structures and three-dimensional menthol centroid coordinates and density maps for open and closed states, with and without PIP_2_, at 279, 290, 300, and 310 K; (4) three-dimensional menthol density maps for open and closed states, with and without PIP_2_, at 279, 290, 300, and 310 K for Y745-neighboring menthol molecules; and (5) data for partition coefficient calculations, theoretical projections, and two-state allosteric model analysis.

## Notes

Details of the CG parametrization of menthol and force field parameters developed (Table S1), mapping scheme, and validation against atomistic reference distributions (Figure S1); backbone root-mean-square deviation (RMSD) of CG TRPM8 simulations (Figure S2); time evolution of menthol partitioning in 10 mM flooding CG simulations (Figure S3); contact probability between menthol and residues lining the orthosteric binding pocket in the VSLD (Figure S4); contact probability between menthol and residues showing meaningful interaction outside the canonical VSLD orthosteric site (Figure S5); spatial distribution of menthol interactions at the TRPM8 Y745 binding site (Figure S6); frequency of menthol interactions with Y745 (Table S2); and theoretical reconstructions of menthol–TRPM8 three-dimensional densities (Figure S7) (PDF)

## References

[1] McGlone F., Reilly D. (2010). The Cutaneous Sensory System. Neurosci. Biobehav. Rev..

[2] McKemy D. D., Neuhausser W. M., Julius D. (2002). Identification of a Cold Receptor
Reveals a General Role for TRP Channels in Thermosensation. Nature.

[3] Peier A. M., Moqrich A., Hergarden A. C., Reeve A. J., Andersson D. A., Story G. M., Earley T. J., Dragoni I., McIntyre P., Bevan S., Patapoutian A. (2002). A TRP Channel
That Senses Cold Stimuli
and Menthol. Cell.

[4] Bautista D. M., Siemens J., Glazer J. M., Tsuruda P. R., Basbaum A. I., Stucky C. L., Jordt S.-E., Julius D. (2007). The Menthol Receptor
TRPM8 Is the Principal Detector of Environmental Cold. Nature.

[5] Colburn R. W., Lubin M. L., Stone D. J., Wang Y., Lawrence D., D’Andrea M. R., Brandt M. R., Liu Y., Flores C. M., Qin N. (2007). Attenuated
Cold Sensitivity in TRPM8 Null Mice. Neuron.

[6] Dhaka A., Murray A. N., Mathur J., Earley T. J., Petrus M. J., Patapoutian A. (2007). TRPM8 Is Required for Cold Sensation in Mice. Neuron.

[7] Tsavaler L., Shapero M. H., Morkowski S., Laus R. (2001). Trp-P8, a Novel Prostate-Specific
Gene, Is Up-Regulated in Prostate Cancer and Other Malignancies and
Shares High Homology with Transient Receptor Potential Calcium Channel
Proteins. Cancer Res..

[8] Zhang L., Barritt G. J. (2006). TRPM8 in Prostate
Cancer Cells: A Potential Diagnostic
and Prognostic Marker with a Secretory Function?. Endocr. Relat. Cancer.

[9] Alaimo A., Genovesi S., Annesi N., De Felice D., Subedi S., Macchia A., La Manna F., Ciani Y., Vannuccini F., Mugoni V., Notarangelo M., Libergoli M., Broso F., Taulli R., Ala U., Savino A., Cortese M., Mirzaaghaei S., Poli V., Bonapace I. M., Papotti M. G., Molinaro L., Doglioni C., Caffo O., Anesi A., Nagler M., Bertalot G., Carbone F. G., Barbareschi M., Basso U., Dassi E., Pizzato M., Romanel A., Demichelis F., Kruithof-de Julio M., Lunardi A. (2024). Sterile Inflammation
via TRPM8 RNA-Dependent TLR3-NF-kB/IRF3 Activation Promotes Antitumor
Immunity in Prostate Cancer. EMBO J..

[10] Ramachandran R., Hyun E., Zhao L., Lapointe T. K., Chapman K., Hirota C. L., Ghosh S., McKemy D. D., Vergnolle N., Beck P. L., Altier C., Hollenberg M. D. (2013). TRPM8 Activation
Attenuates Inflammatory Responses in Mouse Models of Colitis. Proc. Natl. Acad. Sci. U. S. A..

[11] Khalil M., Babes A., Lakra R., Försch S., Reeh P. W., Wirtz S., Becker C., Neurath M. F., Engel M. A. (2016). Transient Receptor Potential Melastatin
8 Ion Channel
in Macrophages Modulates Colitis through a Balance-Shift in TNF-Alpha
and Interleukin-10 Production. Mucosal. Immunol..

[12] Anand U., Korchev Y., Anand P. (2019). The Role of
Urea in Neuronal Degeneration
and Sensitization: An in Vitro Model of Uremic Neuropathy. Mol. Pain.

[13] Lei Z., Ishizuka O., Imamura T., Noguchi W., Yamagishi T., Yokoyama H., Kurizaki Y., Sudha G. S., Hosoda T., Nishizawa O., Andersson K.-E. (2013). Functional Roles of Transient Receptor
Potential Melastatin 8 (TRPM8) Channels in the Cold Stress-Induced
Detrusor Overactivity Pathways in Conscious Rats. Neurourol. Urodyn..

[14] Chasman D. I., Schürks M., Anttila V., de Vries B., Schminke U., Launer L. J., Terwindt G. M., van den Maagdenberg A. M. J. M., Fendrich K., Völzke H., Ernst F., Griffiths L. R., Buring J. E., Kallela M., Freilinger T., Kubisch C., Ridker P. M., Palotie A., Ferrari M. D., Hoffmann W., Zee R. Y. L., Kurth T. (2011). Genome-Wide Association
Study Reveals Three Susceptibility Loci for Common Migraine in the
General Population. Nat. Genet..

[15] Parra A., Madrid R., Echevarria D., Del Olmo S., Morenilla-Palao C., Acosta M. C., Gallar J., Dhaka A., Viana F., Belmonte C. (2010). Ocular Surface Wetness
Is Regulated by TRPM8-Dependent
Cold Thermoreceptors of the Cornea. Nat. Med..

[16] Liu Z., Wu H., Wei Z., Wang X., Shen P., Wang S., Wang A., Chen W., Lu Y. (2016). TRPM8: A Potential
Target for Cancer Treatment. J. Cancer Res.
Clin Oncol..

[17] Behrendt H.-J., Germann T., Gillen C., Hatt H., Jostock R. (2004). Characterization
of the Mouse Cold-Menthol Receptor TRPM8 and Vanilloid Receptor Type-1
VR1 Using a Fluorometric Imaging Plate Reader (FLIPR) Assay. Br. J. Pharmacol..

[18] Bödding M., Wissenbach U., Flockerzi V. (2007). Characterisation of TRPM8 as a Pharmacophore
Receptor. Cell Calcium.

[19] Yang J. M., Li F., Liu Q., Rüedi M., Wei E. T., Lentsman M., Lee H. S., Choi W., Kim S. J., Yoon K. C. (2017). A Novel
TRPM8 Agonist Relieves Dry Eye Discomfort. BMC
Ophthalmol..

[20] Janssens A., Gees M., Toth B. I., Ghosh D., Mulier M., Vennekens R., Vriens J., Talavera K., Voets T. (2016). Definition
of Two Agonist Types at the Mammalian Cold-Activated Channel TRPM8. Elife.

[21] Liu B., Qin F. (2005). Functional
Control of Cold-and Menthol-Sensitive TRPM8 Ion Channels
by Phosphatidylinositol 4, 5-Bisphosphate. J.
Neurosci..

[22] Rohács T., Lopes C. M. B., Michailidis I., Logothetis D. E. (2005). PI­(4,5)­P2
Regulates the Activation and Desensitization of TRPM8 Channels through
the TRP Domain. Nat. Neurosci..

[23] Arcas J. M., González A., Gers-Barlag K., González-González O., Bech F., Demirkhanyan L., Zakharian E., Belmonte C., Gomis A., Viana F. (2019). The Immunosuppressant
Macrolide Tacrolimus Activates Cold-Sensing TRPM8 Channels. J. Neurosci..

[24] Arcas J. M., Oudaha K., González A., Fernández-Trillo J., Peralta F. A., Castro-Marsal J., Poyraz S., Taberner F., Sala S., De La Peña E., Gomis A., Viana F. (2024). The Ion Channel
TRPM8 Is a Direct Target of the Immunosuppressant Rapamycin in Primary
Sensory Neurons. Br. J. Pharmacol..

[25] Gavva N. R., Sandrock R., Arnold G. E., Davis M., Lamas E., Lindvay C., Li C.-M., Smith B., Backonja M., Gabriel K. (2019). Reduced
TRPM8 Expression Underpins Reduced Migraine
Risk and Attenuated Cold Pain Sensation in Humans. Sci. Rep..

[26] Knowlton W. M., Daniels R. L., Palkar R., McCoy D. D., McKemy D. D. (2011). Pharmacological
Blockade of TRPM8 Ion Channels Alters Cold and Cold Pain Responses
in Mice. PLoS One.

[27] Malkia A., Pertusa M., Fernández-Ballester G., Ferrer-Montiel A., Viana F. (2009). Differential Role of the Menthol-Binding
Residue Y745 in the Antagonism
of Thermally Gated TRPM8 Channels. Mol. Pain.

[28] Mälkiä A., Madrid R., Meseguer V., De La Peña E., Valero M., Belmonte C., Viana F. (2007). Bidirectional Shifts
of TRPM8 Channel Gating by Temperature and Chemical Agents Modulate
the Cold Sensitivity of Mammalian Thermoreceptors. J. Physiol..

[29] Chuang H., Neuhausser W. M., Julius D. (2004). The Super-Cooling Agent
Icilin Reveals
a Mechanism of Coincidence Detection by a Temperature-Sensitive TRP
Channel. Neuron.

[30] Andersson D. A., Chase H. W. N., Bevan S. (2004). TRPM8 Activation
by Menthol, Icilin,
and Cold Is Differentially Modulated by Intracellular pH. J. Neurosci..

[31] Brauchi S., Orio P., Latorre R. (2004). Clues to Understanding
Cold Sensation:
Thermodynamics and Electrophysiological Analysis of the Cold Receptor
TRPM8. Proc. Natl. Acad. Sci. U. S. A..

[32] Janssens A., Voets T. (2011). Ligand Stoichiometry of the Cold- and Menthol-Activated Channel TRPM8. J. Physiol.

[33] Matta J. A., Ahern G. P. (2007). Voltage Is a Partial
Activator of Rat Thermosensitive
TRP Channels. J. Physiol..

[34] Taberner F. J., López-Córdoba A., Fernández-Ballester G., Korchev Y., Ferrer-Montiel A. (2014). The Region
Adjacent to the C-End
of the Inner Gate in Transient Receptor Potential Melastatin 8 (TRPM8)
Channels Plays a Central Role in Allosteric Channel Activation. J. Biol. Chem..

[35] Voets T., Droogmans G., Wissenbach U., Janssens A., Flockerzi V., Nilius B. (2004). The Principle of Temperature-Dependent Gating in Cold-
and Heat-Sensitive TRP Channels. Nature.

[36] Choi K. Y., Lin X., Cheng Y., Julius D. (2026). Structural Energetics of Cold Sensitivity. Nature.

[37] Palchevskyi S., Czarnocki-Cieciura M., Vistoli G., Gervasoni S., Nowak E., Beccari A. R., Nowotny M., Talarico C. (2023). Structure
of Human TRPM8 Channel. Commun. Biol..

[38] Yin Y., Wu M., Zubcevic L., Borschel W. F., Lander G. C., Lee S.-Y. (2018). Structure
of the Cold- and Menthol-Sensing Ion Channel TRPM8. Science.

[39] Yin Y., Le S. C., Hsu A. L., Borgnia M. J., Yang H., Lee S.-Y. (2019). Structural Basis
of Cooling Agent and Lipid Sensing
by the Cold-Activated TRPM8 Channel. Science.

[40] Yin Y., Zhang F., Feng S., Butay K. J., Borgnia M. J., Im W., Lee S.-Y. (2022). Activation
Mechanism of the Mouse Cold-Sensing TRPM8
Channel by Cooling Agonist and PIP2. Science.

[41] Yin Y., Park C.G., Zhang F., Fedor J.G., Feng S., Suo Y., Im W., Lee S.Y. (2024). Mechanisms of Sensory Adaptation
and Inhibition of the Cold and Menthol Receptor TRPM8. Sci. Adv..

[42] Bandell M., Dubin A. E., Petrus M. J., Orth A., Mathur J., Hwang S. W., Patapoutian A. (2006). High-Throughput Random Mutagenesis
Screen Reveals TRPM8 Residues Specifically Required for Activation
by Menthol. Nat. Neurosci..

[43] Voets T., Owsianik G., Janssens A., Talavera K., Nilius B. (2007). TRPM8 Voltage
Sensor Mutants Reveal a Mechanism for Integrating Thermal and Chemical
Stimuli. Nat. Chem. Biol..

[44] Becker J., Ellerkmann C. S., Schmelzer H., Hermann C., Lützel K., Gudermann T., Konrad D. B., Trauner D., Storch U., Mederosy (2025). Optical
Control of TRPM8 Channels with Photoswitchable Menthol. Angew. Chem..

[45] Mebrat M. D., Luu D. D., Hilton J. K., Kim M., Parrott K., Cherry B. R., Levitus M., Journigan V. B., Van Horn W. D. (2025). TRPM8 Protein Dynamics Correlates with Ligand Structure
and Cellular Function. J. Am. Chem. Soc..

[46] Chen X., Xu L., Zhang H., Wen H., Yang F. (2022). Differential Activation
of TRPM8 by the Stereoisomers of Menthol. Front.
Pharmacol..

[47] Xu L., Han Y., Chen X., Aierken A., Wang H., Lu X., Zhao Z., Liang P., Yang W., Wen H., Zheng W., Yang S., Yang F. (2020). Molecular Mechanisms
Underlying Menthol Binding and Activation of TRPM8 Ion Channel. Biophys. J..

[48] Bidaux G., Sgobba M., Lemonnier L., Borowiec A.-S., Noyer L., Jovanovic S., Zholos A. V., Haider S. (2015). Functional and Modeling
Studies of the Transmembrane Region of the TRPM8 Channel. Biophys. J..

[49] Pertusa M., Madrid R., Morenilla-Palao C., Belmonte C., Viana F. (2012). *N*-Glycosylation
of TRPM8 Ion Channels Modulates Temperature Sensitivity
of Cold Thermoreceptor Neurons*. J. Biol. Chem..

[50] Pertusa M., Rivera B., González A., Ugarte G., Madrid R. (2018). Critical Role
of the Pore Domain in the Cold Response of TRPM8 Channels Identified
by Ortholog Functional Comparison. J. Biol.
Chem..

[51] Phelps C. B., Gaudet R. (2007). The Role of the N Terminus and Transmembrane
Domain
of TRPM8 in Channel Localization and Tetramerization *. J. Biol. Chem..

[52] Cirqueira L., Stock L., Treptow W. (2022). Concentration-Dependent
Thermodynamic
Analysis of the Partition Process of Small Ligands into Proteins. Comput. Struct. Biotechnol. J..

[53] Ghanakota P., DasGupta D., Carlson H. A. (2019). Free Energies
and Entropies of Binding
Sites Identified by MixMD Cosolvent Simulations. J. Chem. Inf. Model..

[54] Wiley B., Cirqueira L., Naftalin R. J., Domene C. (2026). Atomistic Insights
into Anomeric and Stereochemical Effects on Glucose Transport by GLUTs. J. Am. Chem. Soc..

[55] Treptow W. (2023). Allosteric
Modulation of Membrane Proteins by Small Low-Affinity Ligands. J. Chem. Inf. Model..

[56] Plaza-Cayón A., González-Muñiz R., Martín-Martínez M. (2022). Mutations
of TRPM8 Channels: Unraveling the Molecular Basis of Activation by
Cold and Ligands. Med. Res. Rev..

[57] Luu D. D., Ramesh N., Kazan I. C., Shah K. H., Lahiri G., Mana M. D., Ozkan S. B., Van Horn W. D. (2024). Evidence That the
Cold- and Menthol-Sensing Functions of the Human TRPM8 Channel Evolved
Separately. Sci. Adv..

[58] Sanner M. F., Olson A. J., Spehner J.-C. (1996). Reduced Surface:
An Efficient Way
to Compute Molecular Surfaces. Biopolymers.

[59] Stock L., Hosoume J., Cirqueira L., Treptow W. (2018). Binding of the General
Anesthetic Sevoflurane to Ion Channels. PLoS
Comput. Biol..

[60] Mirdita M., Schütze K., Moriwaki Y., Heo L., Ovchinnikov S., Steinegger M. (2022). ColabFold: Making Protein Folding
Accessible to All. Nat. Methods.

[61] Souza P. C. T., Alessandri R., Barnoud J., Thallmair S., Faustino I., Grünewald F., Patmanidis I., Abdizadeh H., Bruininks B. M. H., Wassenaar T. A., Kroon P. C., Melcr J., Nieto V., Corradi V., Khan H. M., Domański J., Javanainen M., Martinez-Seara H., Reuter N., Best R. B., Vattulainen I., Monticelli L., Periole X., Tieleman D. P., de Vries A. H., Marrink S. J. (2021). Martini 3: A General Purpose Force
Field for Coarse-Grained
Molecular Dynamics. Nat. Methods.

[62] Kabsch W., Sander C. (1983). Dictionary of Protein
Secondary Structure: Pattern
Recognition of Hydrogen-Bonded and Geometrical Features. Biopolymers.

[63] Touw W. G., Baakman C., Black J., Te Beek T. A. H., Krieger E., Joosten R. P., Vriend G. (2015). A Series of
PDB-Related Databanks
for Everyday Needs. Nucleic Acids Res..

[64] Wassenaar T. A., Ingólfsson H. I., Böckmann R. A., Tieleman D. P., Marrink S. J. (2015). Computational
Lipidomics with Insane: A Versatile Tool for Generating Custom Membranes
for Molecular Simulations. J. Chem. Theory Comput..

[65] Pedersen K. B., Ingólfsson H. I., Ramirez-Echemendia D.
P., Borges-Araújo L., Andreasen M. D., Empereur-Mot C., Melcr J., Ozturk T. N., Bennett W. F. D., Kjølbye L. R., Brasnett C., Corradi V., Khan H. M., Cino E. A., Crowley J., Kim H., Fábián B., Borges-Araújo A. C., Pavan G. M., Launay G., Lolicato F., Wassenaar T. A., Melo M. N., Thallmair S., Carpenter T. S., Monticelli L., Tieleman D. P., Schiøtt B., Souza P. C. T., Marrink S. J. (2025). The Martini 3 Lipidome: Expanded
and Refined Parameters Improve Lipid Phase Behavior. ACS Cent. Sci..

[66] Bussi G., Donadio D., Parrinello M. (2007). Canonical
Sampling through Velocity
Rescaling. J. Chem. Phys..

[67] Berendsen H. J. C., Postma J. P. M., van
Gunsteren W. F., Di Nola A., Haak J. R. (1984). Molecular
Dynamics with Coupling to an External Bath. J. Chem. Phys..

[68] Parrinello M., Rahman A. (1981). Polymorphic Transitions
in Single Crystals: A New Molecular
Dynamics Method. J. Appl. Phys..

[69] Humphrey W., Dalke A., Schulten K. V. (1996). Visual
Molecular Dynamics. J. Mol. Graph.

[70] Wan G., Dai X., Yin Q., Shi X., Qiao Y. (2015). Interaction of Menthol
with Mixed-Lipid Bilayer of Stratum Corneum: A Coarse-Grained Simulation
Study. J. Mol. Graphics Modell.

[71] Grünewald, F. ; Marrink, S. J. Fgrunewald/Fast_forward V0.0.1; Zenodo,2022.

[72] Kroon P. C., Grünewald F., Barnoud J., van Tilburg M., Brasnett C., Souza P. C., Wassenaar T. A., Marrink S. J. (2025). Martinize2 and Vermouth Provide a
Unified Framework
for Molecular Topology Generation. Elife.

[73] Abraham M. J., Murtola T., Schulz R., Páll S., Smith J. C., Hess B., Lindahl E. (2015). GROMACS: High Performance
Molecular Simulations through Multi-Level Parallelism from Laptops
to Supercomputers. SoftwareX.

[74] Bernetti M., Bussi G. (2020). Pressure Control Using
Stochastic Cell Rescaling. J. Chem. Phys..

