# Supplementary material for: Temperature-Dependent Menthol Binding across TRPM8 Conformational States
Source: J Am Chem Soc. 2026 Jul 1;148(27):29195–205. doi: 10.1021/jacs.6c07735 (PMC13383620; doi:10.1021/jacs.6c07735)
Supplement: Supplementary file 1 [file ja6c07735_si_001.pdf]

Supporting Information

Temperature-Dependent Menthol Binding Across TRPM8 Conformational States

Leonardo Cirqueira,[1] Guilherme Lucas,[2] and Carmen Domene[1*]

[1] *Departments of Chemistry University of Bath*, Bath BA2 7AX, United Kingdom

[2] Department of Physiology, Ribeirão Preto School of Medicine, University of São Paulo, Ribeirão Preto 14049-900, Brazil

*Corresponding Author: Carmen Domene (C.Domene@bath.ac.uk)

Menthol coarse-grained modeling

A new coarse-grained (CG) model for menthol was developed using the Martini3 force field. [1] The CG representation followed a mapping scheme in which the nonpolar region is described by three small carbon (SC1) beads and the polar region by a single small polar (SP1) bead [2] . This mapping reduces the 31-atom menthol molecule to a four-bead CG representation, as shown in Figure S1. After two rounds of refinement, the resulting CG force field parameters (Table S1) showed good agreement with the atomistic reference distributions, as illustrated in Figure S1.

Table S1. Parameters for coarse-grained menthol following the GROMACS ITP file format. Bond and angle parameters were obtained using the Fast_Forward protocol after initial parametrization and refinement.

Bonds	b_0 (nm)	k_b (kJ mol^{-1} nm^{-2})	Angles	θ_0 (deg)	k_θ (kJ mol^{-1} rad^{-2})
B0-B1	0.244	10,000	B0_B1_B2	157.732	150
B1-B2	0.313	10,000	B0_B1_B3	87.247	50.954
B1-B3	0.282	10,000	B1_B2_B3	53.389	77.822
B2-B3	0.315	10,000			

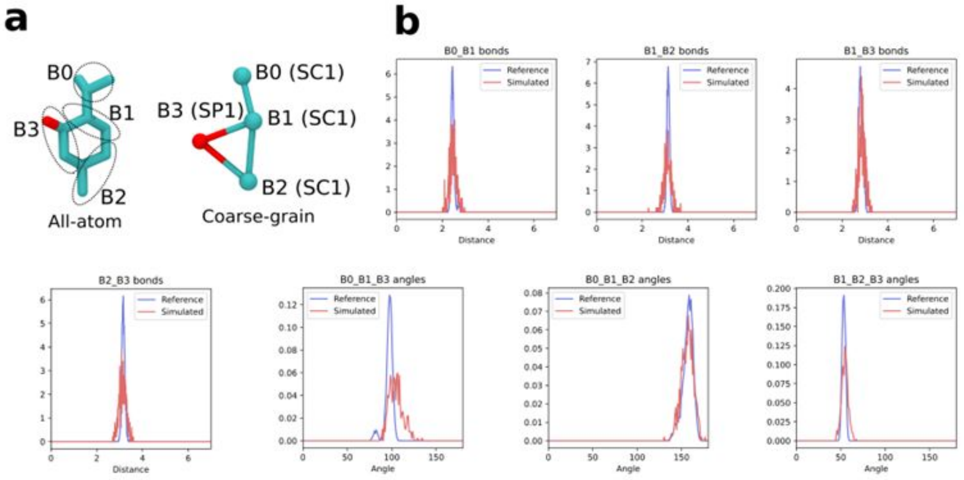

Figure S1. Coarse-grained (CG) parametrization of menthol using Fast_Forward. (a) All-atom to bead mapping for CG menthol. (b) Parametrization results for coarse-grained (CG) menthol in water, comparing atomistic reference distributions (blue) with the corresponding CG distributions (red).

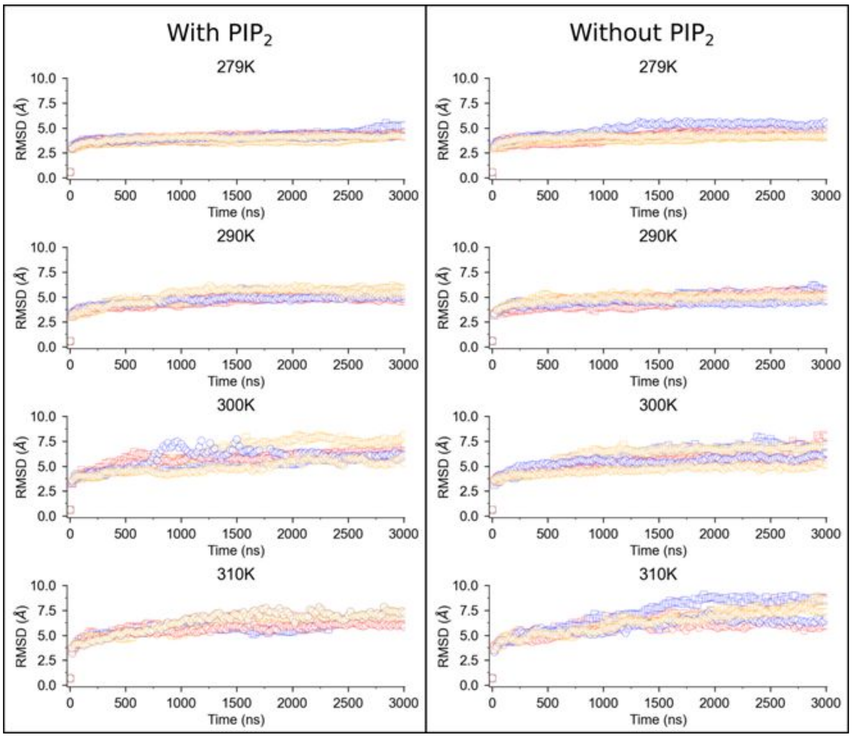

Figure S2. Backbone root-mean-square deviation (RMSD) of CG TRPM8 simulations across temperatures and conformational states. Simulations were performed for both open (circles) and closed (squares) conformations in triplicate (blue, orange, and red) at 279, 290, 300, and 310 K. RMSD values were calculated relative to the first frame.

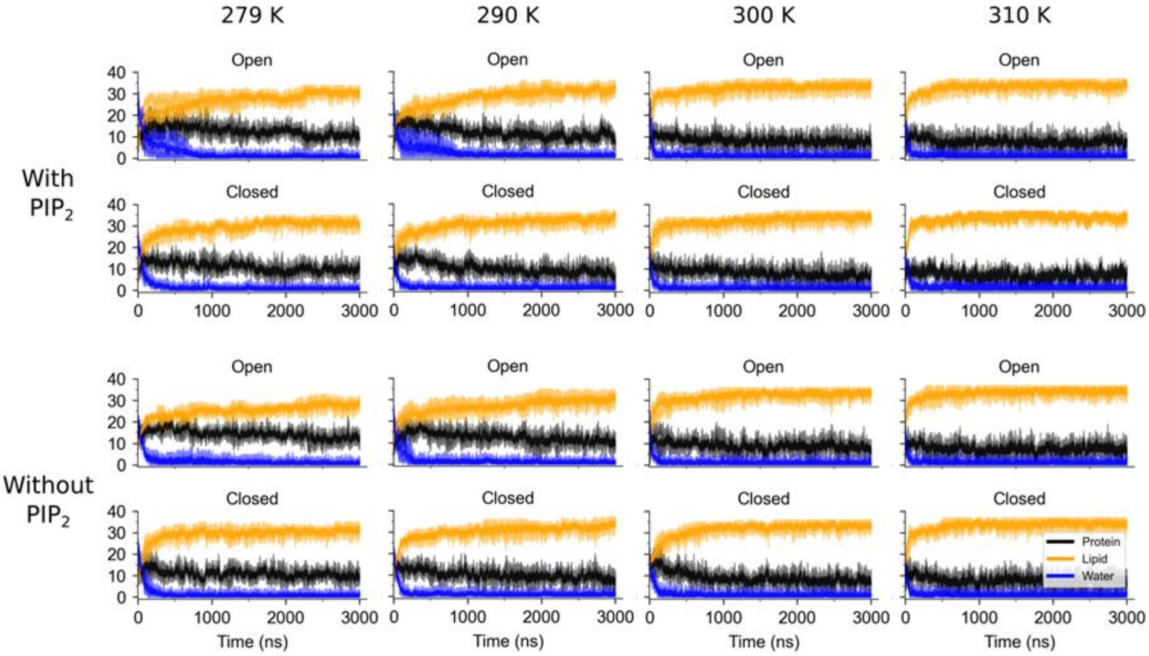

Figure S3. Time evolution of menthol partitioning in 10 mM flooding CG simulations. Ligand counts interacting with protein (black), lipids (orange), and water (blue) are shown for open and closed TRPM8 at 279, 290, 300, and 310 K, with and without PIP$_2$. Protein–ligand interactions were defined using a 5 Å cutoff.

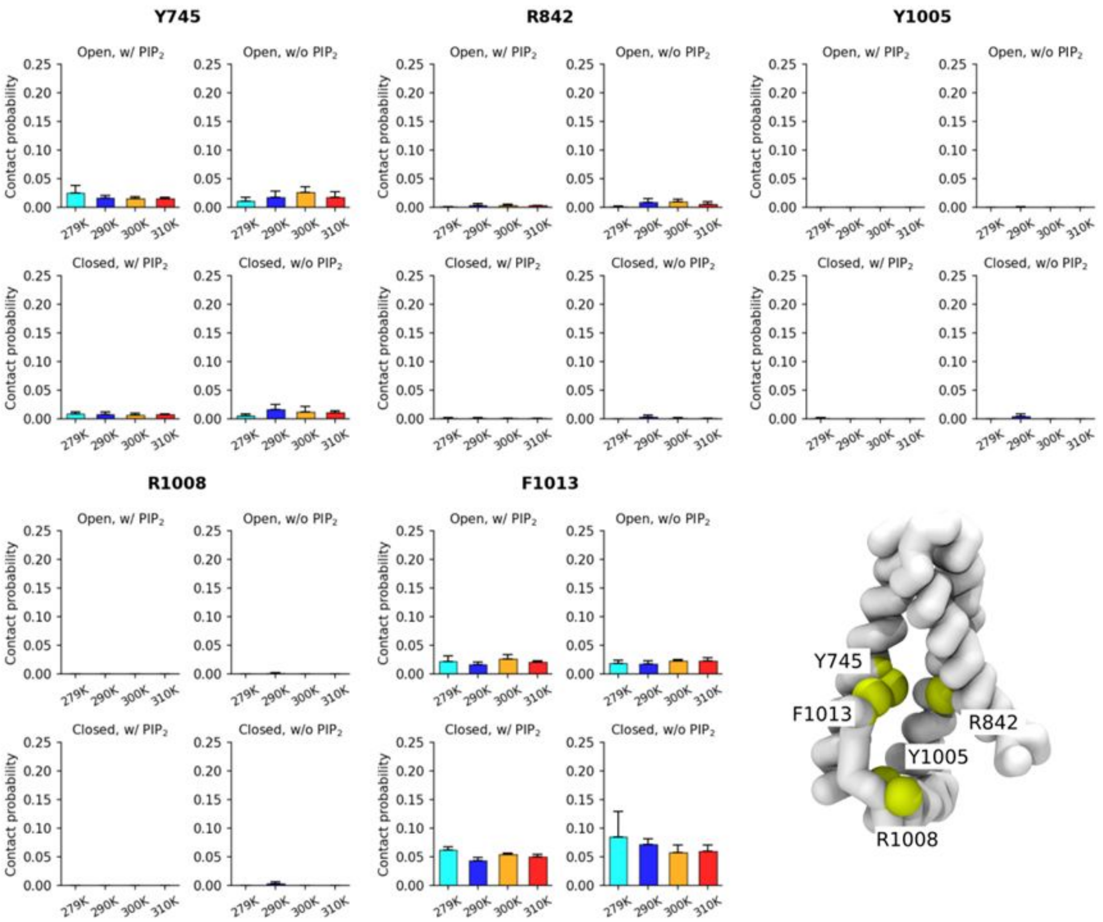

Figure S4. Contact probability between menthol and residues lining the orthosteric binding pocket in the Voltage Sensor-Like Domain (VSLD). Residues were identified from the cryo-EM structure of TRPM8, which resolves the canonical menthol-binding site within the VSLD. Bar plots show the mean contact probability across independent replicas, with and without PIP_2, for open and closed channel conformations at four temperatures (279, 290, 300, and 310 K; cyan, blue, orange, and red, respectively). The inset depicts the VSLD segment in white (S2 helix hidden for clarity), with the five analyzed residues highlighted in yellow.

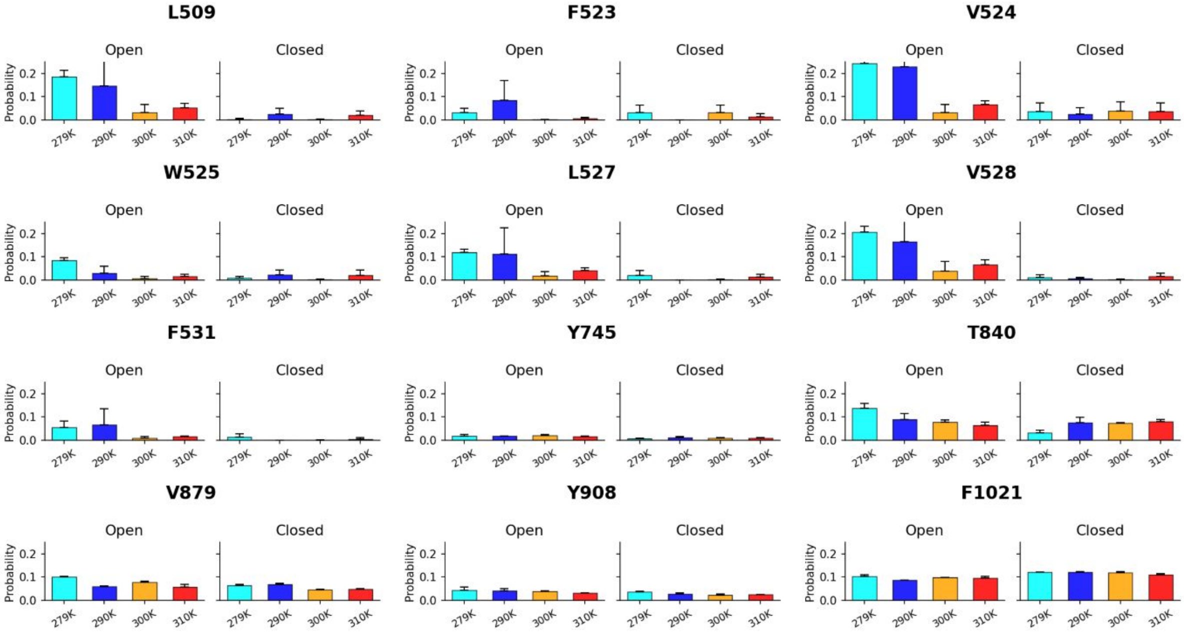

Figure S5. Contact probability between menthol and residues showing meaningful interactions outside the canonical VSLD orthosteric site. Only residues for which sufficient contact events were sampled across the CG trajectories are shown. Bar plots show the mean contact probability across three independent replicas, averaged over systems with and without PIP_2, for open and closed channel conformations separately, at four temperatures (279, 290, 300, and 310 K; cyan, blue, orange, and red, respectively). Residues are grouped by domain: N-terminal region (L509, F523, V524, W525, L527, V528, and F531), pore region (V879 and Y908), and C-terminal region (F1021). For reference, the canonical orthosteric residue Y745 is also included.

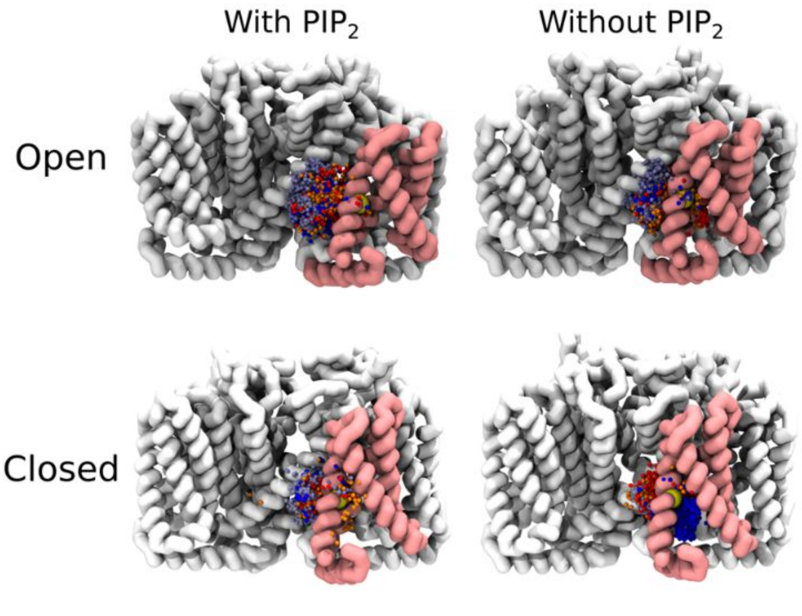

Figure S6. Spatial distribution of menthol interactions at the TRPM8 Y745 binding site in 10 mM CG trajectories. Spheres represent centroid positions of menthol molecules found within 5 Å of residue Y745 (shown in yellow). Data are shown for both open and closed channel conformations. Simulations were conducted at 279 K (purple), 290 K (blue), 300 K (orange), and 310 K (red), with three independent 3000 ns trajectories.

Table S2. Frequency of menthol interactions with TRPM8 residue Y745 in 10 mM flooding coarse-grained (CG) simulations. Simulations were performed in triplicate (3 × 3000 ns) at 279, 290, 300, and 310 K, with and without PIP2. Interactions were counted when any menthol bead was within 5 Å of any Y745 bead.

	With PIP_2		Without PIP_2	
**Temperature (K)**	**Open**	**Closed**	**Open**	**Closed**
279	3%	1%	1%	0%
290	2%	1%	2%	2%
300	2%	1%	3%	1%
310	2%	1%	2%	1%

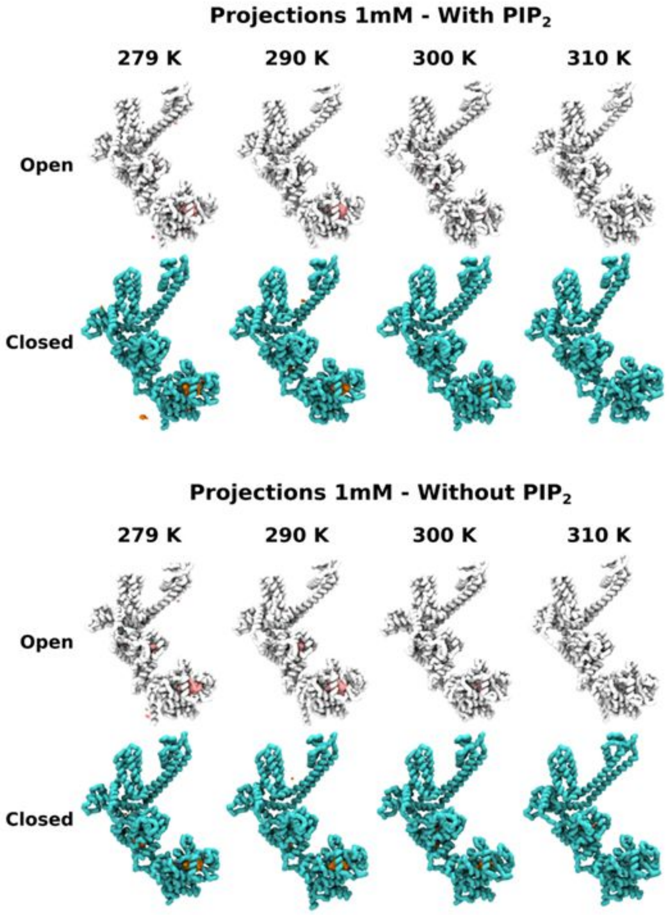

Figure S7. Theoretical reconstructions of menthol–TRPM8 three-dimensional density maps at 1 mM menthol concentration. Densities were reconstructed by rescaling the unitary density map obtained from flooding simulations. Average protein structures are shown for open and closed conformations. The open state is colored white and the closed state cyan, while menthol densities are shown in pink and orange, respectively. A density isosurface cutoff of 3×10^{-4} was used.

References

(1) Souza, P. C. T et al. Martini 3: A General Purpose Force Field for Coarse-Grained Molecular Dynamics. *Nat. Methods* **2021**, *18* (4), 382–388.
(2) Wan, G.; Dai, X.; Yin, Q.; Shi, X.; Qiao, Y. Interaction of Menthol with Mixed-Lipid Bilayer of Stratum Corneum: A Coarse-Grained Simulation Study. *J. Mol. Graph. Model.* **2015**, *60*, 98–107.
